# Complete genome sequences of five *Limnohabitans* strains isolated from two shallow eutrophic lakes in Japan

**DOI:** 10.1128/mra.00944-25

**Published:** 2025-10-29

**Authors:** Rina Kurokawa, Keiji Watanabe, Yusuke Ogata, Shunsuke Takemine, Misa Takagi, Chie Shindo, Wataru Suda

**Affiliations:** 1 RIKEN Center for Integrative Medical Sciences, RIKEN Center for Integrative Medical Sciences 198286, Yokohama, Kanagawa, Japan; 2 Center for Environmental Science in Saitama 290710, Kazo, Saitama, Japan; Montana State University, Bozeman, Montana, USA

**Keywords:** whole genome shotgun, *Limnohabitans*

## Abstract

Freshwater bacterioplankton of the genus *Limnohabitans* are a dominant group with a worldwide distribution. Here, we report the complete genome sequences of five *Limnohabitans* strains: INBF003, INBF004, TEGF002, TEGF003, and TEGF005, isolated from two shallow eutrophic lakes in Japan.

## ANNOUNCEMENT

*Limnohabitans* spp. are cosmopolitan freshwater bacterioplankton that can occur in high abundance (1–3). Here, we report the complete genome sequences of five *Limnohabitans* strains: INBF003 (JCM 16659) and INBF004 (JCM 16660), isolated from Lake Inbanuma, and TEGF002 (JCM 16657), TEGF003 (JCM 16619), and TEGF005 (JCM 16658), from Lake Teganuma in Chiba, Japan. Surface water samples (0–50 cm depth) were collected on 23 January 2008 and filtered through a 0.7 µm glass-fiber filter. The filtrates were spread on modified Reasoner’s 2A (MR2A) agar and incubated at 25°C for 7 days (4). Colonies were subcultured in MR2A liquid medium (pH 7.2), incubated under the same conditions with shaking, and preserved at −80°C in MR2A with 20% glycerol. Genomic DNA was extracted from cultures grown from glycerol stocks.

**Fig 1:**
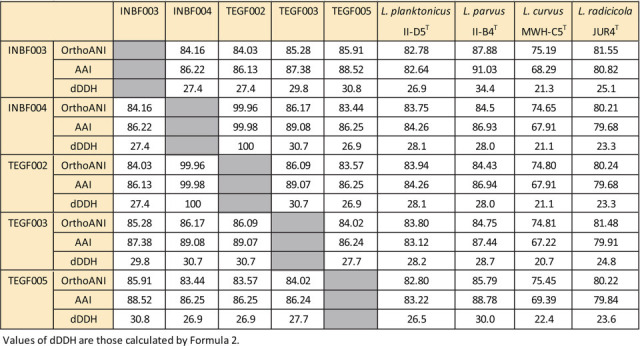
OrthoANI, AAI, and dDDH values for INBF003, INBF004, TEGF002, TEGF003, TEGF005, and four *Limnohabitans* type strains.

Genomic DNA was extracted using enzymatic lysis and phenol–chloroform–isoamyl alcohol extraction, as described previously (5). Whole-genome sequencing was performed using the Sequel II system (PacBio). Genomic DNA was sheared using a g-TUBE (Covaris, Woburn, MA, USA) and a library was prepared with the SMRTbell Express template preparation kit v2.0 (PacBio) without size selection. The PacBio reads were converted to HiFi reads using CCS software v6.2.0. All five strains were assembled using the assembler Canu v.2.1.1 (6) with the parameters -pacbio-hifi, minReadLength = 2200, and minOverlapLength = 2200. Contigs with low depth (<5) were eliminated. The generated contigs were remapped with Minimap2 v2.24-r1122 to check for circularization and to trim terminal overlaps, and the largest circular contig was determined as the chromosome contig. Contigs that mapped to the selected chromosome with identity ≥0.99% and coverage ≥0.95% were considered bubble contigs and subsequently excluded from further analysis. The quality of genome assemblies obtained from strains INBF003, INBF004, TEGF002, TEGF003, and TEGF005 was determined using CheckM v1.1.3 (7), with completeness values of 99.94%, 99.97%, 99.50%, 99.81%, and 99.97%; contamination of 0.26%, 0.55%, 0.55%, 0.73%, and 0.09%; and strain heterogeneity values of 0%, 14.29%, 14.29%, 33.33%, and 0%, respectively. The genomes were annotated and rotated to start at *dnaA* using DFAST v1.2.20 (8). Default parameters were used for all software analysis unless otherwise specified. The obtained reads and genome assemblies are summarized in Table 1, which also includes key assembly statistics (e.g., contig number and genome size). The average nucleotide identity by orthology (OrthoANI) using OAT (9), the average amino acid identity (AAI) values (10), and the digital DNA–DNA hybridization (dDDH) values using the Genome-to-Genome Distance Calculator GGDC 3.0 (11) for INBF003, INBF004, TEGF002, TEGF003, and TEGF005, along with four *Limnohabitans* type strains are shown in Fig. 1.

**TABLE 1:** Summarized reads and contigs data obtained for *Limnohabitans* sp. strains INBF003, INBF004, TEGF002, TEGF003, and TEGF005

Parameter	Data for strain:				
	INBF003	INBF004	TEGF002	TEGF003	TEGF005
Data for quality-checked Sequel reads					
No. of reads	30,000	7,166	7,145	14,354	30,000
Total no. of bases	439,184,207	98,170,221	99,287,384	212,180,912	416,328,792
*N_50_* (bp)	14,626	13,572	13,740	14,640	13,697
Total no. of contigs	1	1	1	1	1
Number of CDSs^*a*^	3,154	3,146	3,149	3,176	2,965
Number of rRNAs	9	9	9	9	6
Number of tRNAs	45	44	44	46	43
Coverage (×)	128	30	31	61	134
BioProject accession no.	PRJDB17422 ^*a*^	PRJDB17422	PRJDB17422	PRJDB17422	PRJDB17422
BioSample accession no.	SAMD00735222	SAMD00735223	SAMD00735224	SAMD00735225	SAMD00735226
Sequence Read Archive (SRA) accession no.	DRR527048	DRR527049	DRR527050	DRR527051	DRR527052
Genome size (bp)	3,381,838	3,174,806	3,173,178	3,400,574	3,050,869
GC content (%)	59.8	59.0	59.0	61.1	59.0
GenBank/ENA/DDBJ accession no.	AP029222	AP029223	AP029224	AP029225	AP029226

^*a*^ CDSs, coding DNA sequences.

Functional annotation revealed that all five *Limnohabitans* isolates possess polyphosphate kinase and creatininase genes, which are associated with phosphate storage and creatinine degradation, respectively.

## Data Availability

The genome sequences and raw sequencing data are available at GenBank/ENA/DDBJ under the BioProject accession number PRJDB17422. Information on the Biosample, DDBJ Sequence Read Archive (DRA), and DDBJ accession numbers is provided in Table 1.

## References

[1] Newton RJ, Jones SE, Eiler A, McMahon KD, Bertilsson S. 2011. A guide to the natural history of freshwater lake bacteria. Microbiol Mol Biol Rev 75:14–49. doi:10.1128/MMBR.00028-10 21372319 PMC3063352

[2] Kasalický V, Jezbera J, Hahn MW, Šimek K. 2013. The diversity of the Limnohabitans genus, an important group of freshwater bacterioplankton, by characterization of 35 isolated strains. PLoS One 8:e58209. doi:10.1371/journal.pone.0058209 23505469 PMC3591437

[3] Jezbera J, Jezberová J, Kasalický V, Šimek K, Hahn MW. 2013. Patterns of Limnohabitans microdiversity across a large set of freshwater habitats as revealed by reverse line blot hybridization. PLoS One 8:e58527. doi:10.1371/journal.pone.0058527 23554898 PMC3595293

[4] Watanabe K, Komatsu N, Ishii Y, Negishi M. 2009. Effective isolation of bacterioplankton genus Polynucleobacter from freshwater environments grown on photochemically degraded dissolved organic matter. FEMS Microbiol Ecol 67:57–68. doi:10.1111/j.1574-6941.2008.00606.x 19049496

[5] Ogata Y, Suda W, Ikeyama N, Hattori M, Ohkuma M, Sakamoto M. 2019. Complete genome sequence of Phascolarctobacterium faecium JCM 30894, a succinate-utilizing bacterium isolated from human feces. Microbiol Resour Announc 8:e01487-18. doi:10.1128/MRA.01487-18 30687834 PMC6346166

[6] Koren S, Walenz BP, Berlin K, Miller JR, Bergman NH, Phillippy AM. 2017. Canu: scalable and accurate long-read assembly via adaptive k -mer weighting and repeat separation . Genome Res 27:722–736. doi:10.1101/gr.215087.116 28298431 PMC5411767

[7] Parks DH, Imelfort M, Skennerton CT, Hugenholtz P, Tyson GW. 2015. CheckM: assessing the quality of microbial genomes recovered from isolates, single cells, and metagenomes. Genome Res 25:1043–1055. doi:10.1101/gr.186072.114 25977477 PMC4484387

[8] Tanizawa Y, Fujisawa T, Nakamura Y. 2018. DFAST: a flexible prokaryotic genome annotation pipeline for faster genome publication. Bioinformatics 34:1037–1039. doi:10.1093/bioinformatics/btx713 29106469 PMC5860143

[9] Lee I, Ouk Kim Y, Park S-C, Chun J. 2016. OrthoANI: an improved algorithm and software for calculating average nucleotide identity. Int J Syst Evol Microbiol 66:1100–1103. doi:10.1099/ijsem.0.000760 26585518

[10] Rodriguez-R LM, Konstantinidis KT. 2014. Bypassing cultivation to identify bacterial species. Microbe Magazine 9:111–118. doi:10.1128/microbe.9.111.1

[11] Meier-Kolthoff JP, Auch AF, Klenk H-P, Göker M. 2013. Genome sequence-based species delimitation with confidence intervals and improved distance functions. BMC Bioinformatics 14:60. doi:10.1186/1471-2105-14-60 23432962 PMC3665452

